# CXCR1/CXCR2 Antagonism Is Effective in Pulmonary Defense against *Klebsiella pneumoniae* Infection

**DOI:** 10.1155/2013/720975

**Published:** 2013-03-18

**Authors:** Jing Wei, Jing Peng, Bing Wang, Hong Qu, Shiyi Wang, Aziz Faisal, Jia-Wei Cheng, John R. Gordon, Fang Li

**Affiliations:** ^1^Department of Immunology, Dalian Medical University, Dalian 116044, China; ^2^Department of Clinical Laboratory, Karamay Central Hospital, Karamay 834000, China; ^3^Department of Biochemistry, Dalian Medical University, Dalian 116044, China; ^4^Department of Life Science, Institute of Biotechnology, National Tsing Hua University, Hsinchu 300, Taiwan; ^5^The Division of Respirology, Critical Care and Sleep Medicine, Royal University Hospital, University of Saskatchewan, Saskatoon, SK, Canada S7N 0W8

## Abstract

*Klebsiella pneumoniae*-associated pathology is largely mediated by neutrophilic inflammation. In this study, we administered *Klebsiella pneumoniae* to experimental guinea pig groups and the ELR-CXC chemokine antagonist CXCL8_(3–72)_, ceftazidime, and dexamethasone to different groups, respectively. After 24 h, we assessed the animal's pulmonary inflammatory levels, including gross histopathology, airway neutrophilia, lung myeloperoxidase levels, expressions of CXCL8 and TNF, and airway bacterial loads. Compared with ceftazidime and dexamethasone treatments, the administration of the ELR-CXC chemokine antagonist CXCL8_(3–72)_ alone was more effective than other methods, although it did not markedly attenuate the bacterial load. These results suggest new methods for the treatment of *Klebsiella pneumoniae* pathology.

## 1. Introduction

*Klebsiella pneumoniae*, a human intestinal flora, is the second most common cause of bacteremia which is frequently associated with hospital-acquired infections such as malignancy, cirrhosis, biliary tract disorders, and diabetes mellitus (DM) [1]. It accounts for a significant proportion of hospital-acquired urinary tract, *pneumonias*, and soft tissue infections, partly because of the frequent spreading of these organisms in hospital environments [2]. Pyogenic brain abscess, meningitis, and endophthalmitis, which are related to metastatic infections, are the most important characteristics of *Klebsiella pneumoniae* infections [1]. Lipopolysaccharide (LPS) is considered to be a major virulence determinant of *Klebsiella*, and mutations leading to lipid A anchor of its LPS are conditionally lethal and result in the enhancement of lethality [3]. Multidrug resistant *Klebsiella pneumoniae* organisms can cause nosocomial outbreaks, which are called extended-spectrum-beta-lactamase- (ESBL-) producer bacteria. The incidence of ESBL-producing strains has been steadily increasing over the past years thus limiting our therapeutic options because of the increase of drug resistance [4]. New and alternative measures are required to control further *Klebsiella*-associated hospital infections and their multidrug resistance. Chemokines and chemokines receptors are attractive pharmacological target for the treatment. They are secreted locally or in paracrine and autocrine fashions by leucocytes and tissue cells. They activate a G protein-coupled receptor which is involved in the attraction of leukocytes. The CXC chemokines (alpha chemokines) are classified into a subgroup dependent on the presence of the Glu-Leu-Arg (ELR) motif. This motif is conserved in all natural ligands of CXCR1, and CXCR2, and is essential for their activation by chemokines. Neutrophil recruitment is regulated by ELR-CXC and their activation is induced by the engagement of CXCR1 and/or CXCR2 [5]. CXCL8 (interleukin; (IL-8)), the prototypical ELR-CXC chemokine, is frequently detected in microbial infections, including *Klebsiella* infections [6]. CXCR1 acts as a receptor for CXCL8 and CXCL6 (granulocyte chemoattractant protein-2), while CXCR2 also binds with other ELR-CXC chemokines including those chemokines highlighted earlier. Compared to other chemokines, CXCL8 has a high affinity at these receptors [5]. ELR-CXC chemokines and other inflammatory mediators are mainly expressed by endothelial cells following bacterial activation in the airways [7, 8]. CXCR2 alone can produce a reduction in neutrophil infiltration, whilst CXCR1, and CXCR2, in combination, can regulate the neutrophil response to ELR chemokines. J. R. Gordon reformed human CXCL8 cDNA. They mutated lysine to arginine in the 11th position and glycine to praline in the 31th position. Then they got high-binding affinity without chemotactic activity product, named CXCL8_(3–72)_ K11R/G31P (G31P in abbreviation). G31P is highly effective in blocking neutrophil recruitment into both microbial and nonmicrobial inflammatory responses [8–10].

## 2. Materials and Methods

### 2.1. Reagents and Bacteria

The following reagents were purchased: myeloperoxidase test kit (Nanjing, China), TRIzol RNA extraction kit, RT-PCR kit, and agarose (Dalian Takara Bio Inc., Dalian, China).The* Klebsiella pneumoniae *strain was donated by Ms. J. Wang of the First Affiliated Hospital of Dalian Medical University Laboratory. The concentration of bacteria was 10^9^ cfu/mL (UV at Abs_600_). CXCL8, K11R/G31P chemokines were expressed and purified in our laboratory [8].

### 2.2. Guinea Pig Klebsiella pneumoniae Model

Six-week old guinea pigs of female gender and weighing about 250 g were obtained from the Dalian Medical University Laboratory Animal Center. All protocols followed the ethics guidelines regarding the treatment of animals used in experiments. We divided the animals into batches of five groups (*n* = 5 per group). The animals were anaesthetized with pentobarbital sodium (50 *μ*g/kg). Group 1 guinea pigs were inoculated intraperitoneally with 10^9^ cfu/mL of *K*. *pneumoniae* 200 *μ*L (diluted with saline) and are referred to as the controls. Other three groups (from Group 1 to Group 4) were experimental groups which were given *Klebsiella pneumoniae* and different treatments (30 minutes before infection). Group 2 guinea-pigs were administered with G31P (500 *μ*g/kg; i.p.), Group 3 with ceftazidime (200 mg/kg; s.c.), and Group 4 with dexamethasone (0.8 mg/kg; s.c.). Group 5 guinea-pigs were given saline only (s.c.). Animals were harvested 24 h after the bacterial/sham procedures. All experiments were repeated three times, with the depicted data being representative.

### 2.3. Myeloperoxidase Assay (MPO)

To determine myeloperoxidase (MPO) activity, BALF was incubated with o-dianisidine dihydrochloride in Hank's medium supplemented with 0.004% H_2_O_2_. The reaction was stopped by adding 1% NaN_3_, and MPO activity was determined by spectrophotometry(OD 460 nm).

### 2.4. Isolation of Total RNA from Lung Tissue

Lung tissue RNA was isolated using a TRIzol RNA purification kit, according to the supplier's instructions. Semiquantifications and purity assessments were performed by optical density (OD) measurements at 260 nm and 280 nm and samples stored at −80°C.

### 2.5. Reverse Transcriptase-Polymerase Chain Reaction (RT-PCR)

Total RNA was extracted with TRIzol reagent according to the manufacturer's protocol. Semi-quantifications and purity assessments were performed by optical density (OD) measurements at 260 nm and 280 nm. Total RNA was reverse transcribed into cDNA using an RNA PCR Kit (see the details in Table 1).

The first steps of the RT condition were 53°C for 30 min, 99°C for 5 min, and 5°C for 5 min. *β*-actin PCR reactions were performed as follows: initial denaturation at 94°C for 2 min, 30 cycles at 94°C for 30 s, 58°C for 30 s, and 72°C for 2 min, and a final extension at 72°C for 10 min. Other cytokines PCR reactions were performed as follows: initial denaturation at 94°C for 2 min, 30 cycles of 94°C for 30 s, 57°C for 30 s, 72°C for 2 min, and a final extension at 72°C for 10 min. The subsequent results were analysed using 1% agarose gel and a gelatinous imaging system in order to detect the integral absorbance (IOD).

### 2.6. Bronchoalveolar Lavage (BAL) Neutrophil Counting

Each guinea-pig was anesthetized by intraperitoneal injection of ketamine/xylazine. The trachea was exposed, and a bronchoalveolar lavage (BAL) was performed with 1 mL sterile PBS. BAL fluid (BALF) was subjected to 10-fold serial dilutions. Neutrophils were counted using a microscope. 

### 2.7. Statistical Analyses

Statistical analysis of the data and group comparisons were made using a Student's *t*-test (two-tailed). Statistical significance was assigned with *P* ≤ 0.05. The results are expressed as the mean ± SEM.

## 3. Results

### 3.1. ELR-CXC Chemokine Antagonism Significantly Decreases Airway Neutrophil Recruitment and Ameliorates Pulmonary Level Inflammation

ELR-CXC chemokine antagonists play an effective role in airway neutrophil recruitment and exert an inflammatory action. We assessed their pulmonary anti-inflammatory effects 24 hours after giving bacteria. We found the lungs to be pink, soft to touch, and with no evidence of inflammatory cell infiltration in normal animals. The lungs of the *Klebsiella*-treated animals presented with substantial pleural hemorrhagia, the airways were congested with inflammatory cells, and the lung parenchyma was edematous. The lungs of the G31P-treated group exhibited light pleural pathology, and the airways and lung parenchyma displayed mild edema with few inflammatory cells present.

### 3.2. Bronchoalveolar Lavage (BAL) Analyses by MPO Assay

**Figure 1:**
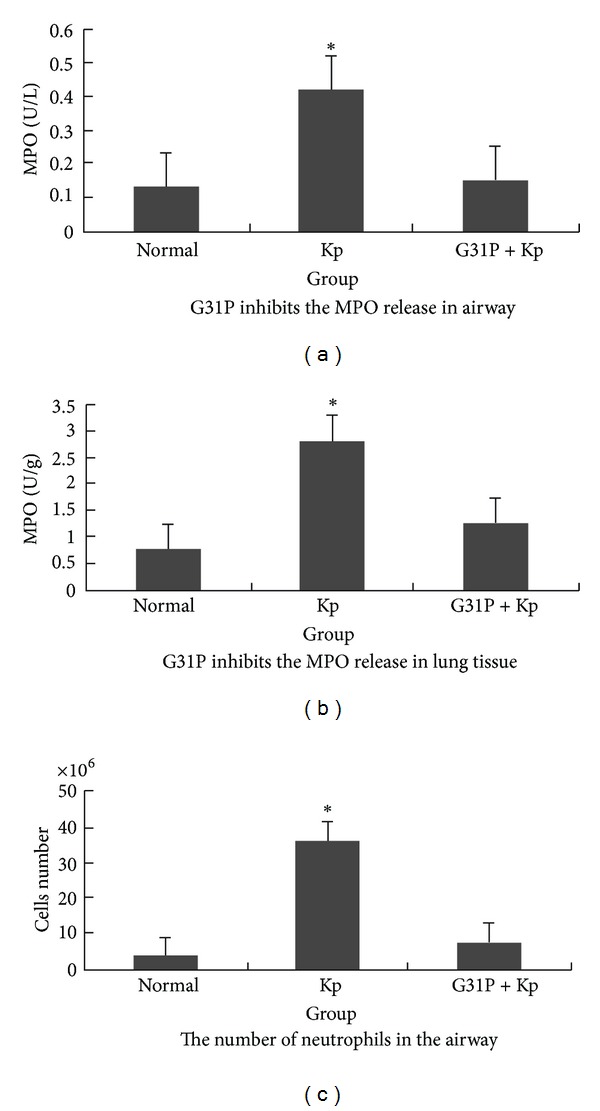
Treatment with the ELR-CXC chemokine antagonist G31P reduces pulmonary neutrophil responses in guinea pigs with *Klebsiella pneumoniae*. Guinea pigs were challenged with *Klebsiella pneumoniae* and treated with G31P. The airway neutrophil responses were assessed from differential counts of cells recovered by bronchoalveolar lavage (BAL). The airway (BAL fluid) levels of myeloperoxidase (MPO), as surrogate measurements of neutrophilic inflammation, were assessed using a chromogenic assay. The G31P treatments reduced each of these indicators of neutrophilic inflammation close to normal. This experiment was repeated three times; the data depicted are representative of each group.

Neutrophil analyses were carried out by determining MPO activity. Group-1 animals contained about 37% neutrophils, while Group-2 contained only background numbers of neutrophils (*P* > 0.05 versus the normal control animals). BAL fluid MPO levels mirrored these results. The G31P treatments significantly reduced the MPO levels to near baseline (*P* > 0.05 versus the normal control animals, see Figure 1). Meanwhile, compared with the control group, G31P markedly reduced the expression levels of the inflammatory mediators IL-1, CXCL8, and TNF. We thus observed a guinea pig model of aspiration *pneumoniae* [11] and amelioration of the neutrophil responses of pneumonic guinea-pigs following the administration of G31P, which did not affect their ability to resist a *Klebsiella* infection.

### 3.3. ELR-CXC Chemokine Antagonism Is as Effective as Corticosteroid and Antibiotic Treatments

**Figure 2:**
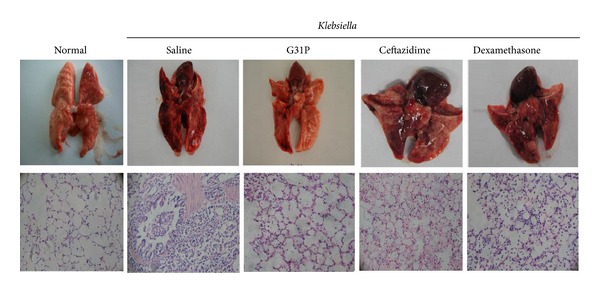
Comparison of different treatments for inflammatory pathology in guinea pigs with *Klebsiella pneumoniae*. The lungs of the control group were normal in gross appearance, while the lungs of the bacterial control group were markedly hemorrhagic and congested. The G31P-treated group was largely free of hemorrhagic consolidation and had greatly reduced pulmonary inflammation. The lungs of the saline controls exhibited a normal histological appearance, while the lungs of the bacterial control animals were markedly hemorrhagic and congested with red blood cells (RBC) and neutrophils (PMN).

**Figure 3:**
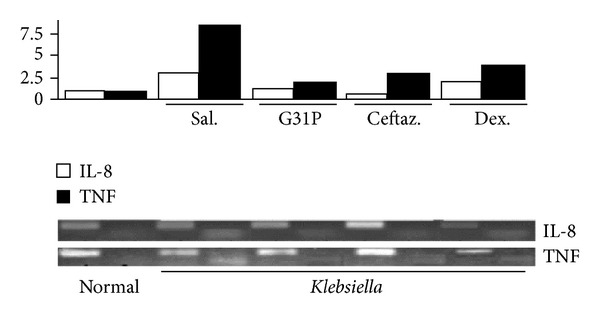
Impact of G31P, dexamethasone, or ceftazidime treatments on inflammatory pathology in guinea pigs with *Klebsiella pneumoniae*. Guinea-pigs were challenged with *Klebsiella pneumonia*, treated with G31P, dexamethasone, or ceftazamine, and then sacrificed 24 h later. Each of these treatments markedly reduced the expression levels of both mediators relative to the saline-treated animals. Each experiment was repeated three times; the data depicted are representative of each group.

**Figure 4:**
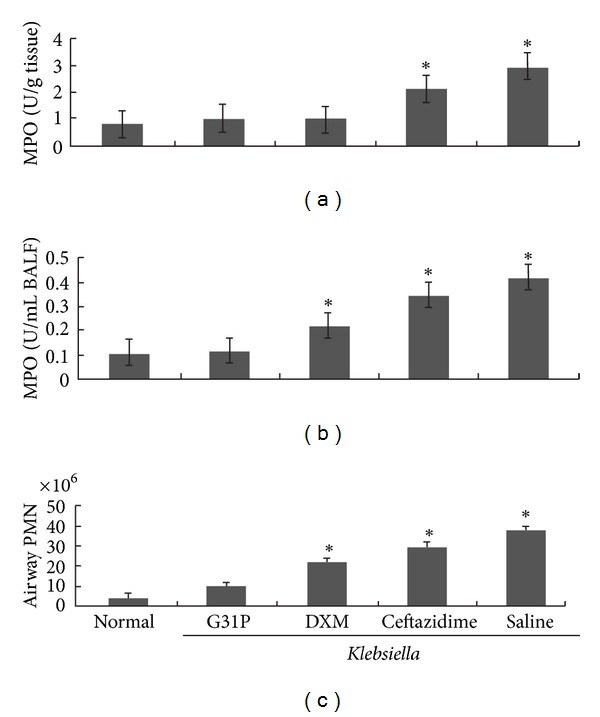
Impact of G31P, dexamethasone, or ceftazidime treatments on inflammatory pathology in guinea pigs with *Klebsiella pneumoniae*. In the G31P-treated group, PMN decreased significantly in BALF compared with the hormones, or antibiotics-treated group (*indicates *P* < 0.05). The data show that G31P, hormones, and antibiotics groups have a significant effect in the inhibition of MPO release from lung tissue (*indicates *P* < 0.05). G31P decreased significantly the release of MPO in lung tissue compared with the antibiotics group (*indicates *P* < 0.05). But there was no significant difference between the G31P and DXM groups.

Either antagonizing the bacterial growth or dampening the inflammatory response pharmacologically has significant protective effects in the antimicrobial inflammatory response of bacterial *pneumonias *[12, 13]. We compared curative effects of different treatments, including G31P, the broad-spectrum *β*-lactam antibiotic ceftazidime (200 mg/kg), and dexamethasone (0.8 mg/kg/24 h). 24 h posttreatment, ceftazidime reduced inflammation levels but had no effect in eliminating the pulmonary *Klesbiella* load, pleural hemorrhagic consolidation (Figure 2), or CXCL8 and TNF responses (Figure 3). And it had marginal effects in preventing white cell infiltration (Figure 4). The dexamethasone treatment had no significant effects on airway *Klebsiella* loads, but it significantly improved pathologic damage (Figure 2), CXCL8 and TNF (Figure 3) levels, infiltration of inflammatory cells, and the lung MPO response (Figure 4). G31P treatment was more effective in reducing neutrophilic pathology (Figure 2) than antibiotic and corticosteroid treatments. CXCL8 and TNF levels reduced (Figure 3) airway inflammatory cell influx, MPO levels, and lung parenchymal MPO levels (Figure 4). 

## 4. Discussion

Chemokines are small molecular proteins (8~10 KDa) that are important in the recruitment of leukocytes to extravascular tissues [6]. Although this is a normal physiological response during infection, excessive inflammation cells can cause tissue injury. In such cases chemokine antagonism can be used as a therapeutic strategy. 

The ELR^+^ CXC chemokines include CXCL8, epithelial neutrophil activator 78 (ENA-78; CXCL5), granulocyte chemotactic protein-2 (GCP-2; CXCL6), neutrophil-activating protein-2 (NAP-2; CXCL7), and growth-related oncogene-*α*, -*β*, and -*γ* (GRO-*α*, -*β*, and -*γ*, resp. CXCL1, CXCL2, and CXCL3) [5]. These chemokines can bind to either CXCR1 or CXCR2, and recruit neutrophils into the local inflammatory response [2] in a complex multistep process [14–16]. The CXCR1 binds CXCL8 and GCP-2 with high affinity, while the CXCR2 binds CXCL8 with higher affinity, but other ELR-CXC chemokines at low affinity [5]. We previously engineered a human CXCL8 analogue (CXCL8_(3-72)_ K11R/G31P; G31P) [17]. G31P antagonizes both CXCL1 and CXCL2 efficiently, but also desensitizes heterologous G protein-coupled receptors (GPCR) on cells that express CXCR1 or CXCR2 (i.e., the receptors for C5a, LTB4, and fMLP) [8, 17]. It has been reported that G31P serves as an effective anti-inflammatory agent in models of airway endotoxemia [9, 10, 18], aspiration *pneumoniae* [11], and ischemia-reperfusion injury [10, 19]. It has been reported previously that bacterial endotoxin-challenged bronchial epithelial cells secrete high levels of CXCL8 and CXCL5 and that these act as autocrine agonists for these cells [8], and others have documented that vascular endothelial and alveolar epithelial cells expression of CXCR2 plays an important role in pulmonary pathology in models of LPS-induced *pneumoniae* in mice [20, 21]. Specifically, G31P therapy reduced pulmonary expression of endogenous pyrogens (TNF and IL-6) as well as CXCL8, and this was associated with a dramatically reduced neutrophilic inflammatory response, but no change in the pulmonary loads of viable *Klebsiella pneumoniae*. Corticosteroids can be used effectively to dampen inflammation, but there is some discrepancy regarding whether they antagonize ELR-CXC chemokine responses [22–24] or not [25, 26] and thereby antagonize neutrophil responses. Nevertheless, the lung tissues of dexamethasone-treated mice contained reduced levels of TNF, CXCL8, and MPO and modestly reduced numbers of neutrophils relative to the saline-treated pneumonic animals, although the levels of each of these parameters still appeared to be somewhat higher than those seen in G31P-treated mice.

Aggarwal et al. reported a metastatic role of CXCL8, which promotes cell growth and metastasis. CXCL8 expression correlates with metastatic potential in human melanoma and ovarian cancer cells. It has been found to play an important role in astrocytomas, anaplastic astrocytomas, glioblastomas, central nervous system cervical carcinoma metastasis, brain tumors, gastric cardia adenocarcinoma (GCC), and esophageal squamous cell carcinoma [6]. G31P can be used as an antagonistic agent to treat the carcinoma by reducing the level of CXCL8. This study opens the future research aspect of ELR-CXC to treat these carcinomas.

In summary, our data indicate that the ELR-CXC antagonist G31P can effectively reduce *Klebsiella pneumoniae* pathology in a guinea-pig model. It is suggested that broad-spectrum ELR-CXC chemokine antagonism may be a useful adjunct in the therapeutic approach to treat *Klebsiella pneumoniae* in patients.

## Figures and Tables

**Table 1:** The sequence of primers and the length of amplification products.

Name	Primer sequence	Length of product (bp)
*β*-actin	Forward 5-CGTAAGGACCTCTATGCCAACAC-3 Reverse 5-GACTCATCGTACTCCTGCTTGCT-3	246
CXCL8	Forward 5-TGCGATGCCAGTGTATTAAGATT-3 Reverse 5-CTCTTGAAGAACATGCTCACCAC-3	94
TNF	Forward 5-CACTCATCTACTCCCAGGTCCTC-3 Reverse 5-ACTCTTGATGGCAGAGAGAAGGT-3	158
IL-1*α* (CXCL1)	Forward 5-CTTCTGCCATTGACCATCTCTCT-3 Reverse 5-TCTGAGTTTTCGTAGGGATTCGG-3	126

bp: base pair; IL: interleukin; CXCL1: chemokine (C-X-C motif) ligand 1; TNF: tumor necrosis factor.
